# Establishing an online physical exercise program for people with hemophilia

**DOI:** 10.1007/s00508-019-01548-1

**Published:** 2019-09-18

**Authors:** Barbara Wagner, Axel Seuser, Steffen Krüger, Marie Luca Herzig, Thomas Hilberg, Cihan Ay, Timothy Hasenöhrl, Richard Crevenna

**Affiliations:** 1 Department of Physical Medicine, Rehabilitation and Occupational Medicine, Medical University of Vienna, Waehringer Guertel 18–20, 1090 Vienna, Austria; 2 Practice for Rehabilitation, Prevention and Orthopedics, Hans-Böckler-Straße 19, 53225 Bonn, Germany; 3 Department of Sports Medicine, University of Wuppertal, Moritzstraße 14, 42117 Wuppertal, Germany; 4 Department of Medicine I, Division of Hematology and Hemostaseology, Medical University of Vienna, Waehringer Guertel 18–20, 1090 Vienna, Austria

**Keywords:** Hemophilia, Haemophilia, Exercise, Training, Rehabilitation, Home-based

## Abstract

**Background:**

Hemophilia is a congenital bleeding disorder with an estimated frequency of 1:10,000 births. Repeated joint bleeding is a hallmark of the disorder and leads to painful hemophilic arthropathy. Regular exercise can help improve joint stability and function, reduce the risk of injury and bleeding and improve physical fitness and quality of life. This method paper describes an online training concept aiming to offer access to appropriate exercise instructions for people with hemophilia who are not able to attend regular training at a hemophilia center.

**Methods:**

The online exercise program is accessible through the homepage of the Department of Physical Medicine, Rehabilitation and Occupational Medicine of the Medical University Vienna as well as through scanning a QR code printed on information material using a smart phone or tablet.

**Results:**

The program contains exercises to improve mobility, coordination, muscular strength and flexibility. A brief introduction is given by a hematologist, a pediatrician and a physiatrist. An introductory video informs about contraindications and essential precautions, such as medical attendance and sufficient factor therapy to consider before starting the training. Another video gives advice on the exercise composition. The demonstrated exercises are explained by a physician and are available for adults and children. To individualize training recommendations and offer further diagnostic tools and physical treatment options as necessary, the Department of Physical Medicine, Rehabilitation and Occupational Medicine of the Medical University of Vienna will establish consultation hours for people with hemophilia.

**Conclusion:**

As hemophilia is an orphan disease, patients are mainly treated in specialized centers. For patients who live far from these centers or have limited access to a training there for other reasons, the physical medicine consultation hour and the implementation of online exercise instructions offer individually adapted exercise information for a regular home-based training to benefit from increased physical fitness and joint stability.

## Introduction

### Musculoskeletal complications in hemophilia

Hemophilia is an X‑chromosome linked bleeding disorder caused by a deficiency of coagulation factor VIII (in hemophilia A) or factor IX (in hemophilia B) with an estimated frequency of 1:10,000 births [1, 2]. Currently, the Austrian hemophilia registry lists 753 patients [3].

Hemorrhages mainly affect joints (ankles, knees, elbows) and muscles [2]. Blood and inflammatory enzymes together with impaired joint loads cause articular damage and a progressive arthritic condition [1, 2, 4–6]. At a very early stage of illness, preclinical signs can be detected using thermography and silent symptoms (tender points) [7]. Recurrent hemarthrosis may lead to chronic hemophilic arthropathy associated with strength deficits, muscle atrophy, contractures, pain-related alterations of intramuscular and intermuscular coordination, joint instability and angular deformities and may finally result in joint ankylosis, disability and a diminished quality of life [1, 8–15, 79]*.*

### Deficits in the basic motor functions in people with hemophilia

Several studies have found deficits in the basic motor functions in people with hemophilia (PwH) [16–22]. Deficits regarding coordination can be found in children and adults [16, 20]. Adults also show significant impairments in dynamic balance and proprioception [23, 24]. Several studies have found diminished strength for certain muscle groups and patient characteristics (depending on the age, joint status and severity of the disease), showing that hemophilic arthropathy is accompanied with deficits in the upper and lower limbs and back muscles [9, 13, 19, 23, 25, 26]. Studies on children and adolescents have shown contradictory results regarding strength performance. A main problem seems to be the muscles surrounding the knee and elbow joints depending on the joint condition [26–31, 79]. Seuser et al. found a significantly reduced ratio between back and abdominal strength [16] and PwH also show impaired muscle function of the lower limbs regardless of overt joint affection [32, 33]. Seuser et al. found a reduced passive mobility of the ischiocrural muscles in children and adolescents [16] and Czepa et al. demonstrated a diminished joint range of motion for the elbows, knees and ankles in adult PwH [20]. Studies have shown that adult as well as young PwH have lower maximum and submaximum endurance performance compared to healthy controls [16, 21, 28].

### Training components, aims and benefits for people with hemophilia

In a healthy population, improved fitness in endurance as well as muscular strength is associated with significantly better cardiometabolic risk factor profiles, reduced general mortality, a lower risk of developing functional limitations and possibly less symptoms of depression and fatigue [34]. Resistance exercise improves bone mass and can reduce pain and disability in patients with osteoarthritis [34]. In the field of hemophilic arthropathy, resistance exercise together with coordination and endurance training is important to improve joint stability and to control exaggerated end-range of motion joint movements, thereby reducing the risk of injuries, falls and hemarthroses [1, 9, 13–15, 17–19, 35–42, 79]. According to the World Federation of Haemophilia guidelines [1], adapted physical activity with a focus on strength, coordination, physical function and overall fitness is therefore recommended [1, 43].

Exercise programs for hemophilia patients consist of several components that may be most beneficial when applied together: flexibility and stretching (including a warm up before activity starts to reduce the risk of injury), strength, sensorimotor training (or proprioception), balance as well as overall function [38, 44]. Flexibility and stretching increase the range of motion and help reduce tension in skeletal muscles [44]. Increased joint and core muscle strength helps to control exaggerated end-range of motion joint movements and therefore can help to prevent or decrease synovial impingement and associated hemarthroses or synovitis and reduce pain [44]. Sensorimotor and balance training promote joint stability and function [44]. Specific treatment objectives of physical therapy include the reduction of pain, the prevention of muscle atrophy, an increase in range of motion, an improvement in functional ability, a reduction of joint bleeding and an improvement in quality of life [44, 45]*.*

### Aim of establishing an online exercise protocol for people with hemophilia

As hemophilia is a complex and rare disorder (orphan disease), PwH are mainly treated in hemophilia treatment centers that bring together healthcare professionals experienced in treating PwH [46]. To improve and maintain physical fitness it is essential to perform cardiorespiratory, resistance, flexibility and neuromotor exercise training on a regular basis [34]. For PwH who live far from these centers, the travel distance and time, limited public transportation and travel costs as well as reduced mobility due to the arthropathy may represent a considerable barrier for a regular participation in physical therapy and training guided by musculoskeletal specialists at a medical center. These PwH might also benefit from the program if they have little spare time due to personal, private and job-related circumstances. The new generation likely appreciates the use of new media for additional training advice. This method paper describes a new training concept aiming to promote the inclusion and participation of PwH in physical activity. The online videos aim to enable PwH to participate in an easily accessible, time-saving, free of charge and disease-adapted training to gain from the multiple benefits of increased physical fitness.

## Methods

Implementing the online training program had the following requirements:

- The chosen exercises should be safe when applied correctly
- The exercises should be practicable for most of the PwH including patients who already suffer from arthropathic joint conditions
- The training should be effective and should consider the most important deficits in PwH
- As the videos are used by lay persons, instructions on how to choose, compose and perform the exercises should be simple and comprehensible
- Patients should not need to buy expensive training equipment, this is why resistance exercises using body weight and therabands were chosen
- For adults who have already developed chronically affected joints there should be the possibility to specifically focus the training on these joints
- As training is only effective if it is adapted over time according to the increasing fitness, the possibility to increase the training has to be part of the program.

The concept of the online training program was compiled in accordance with musculoskeletal experts in the field of hemophilia from Germany. The videos were recorded separately for adults and children. The video clips were shot with a person with hemophilia and the background of studying sports science (who demonstrated the exercises for the adult videos) and a 10-year-old volunteer (who demonstrated the exercises for the children videos). During the takes a physiotherapist was present to observe the correctness of the exercise performance.

There are three options to access the webcasts:

- via links listed on the homepage of the Department of Physical Medicine, Rehabilitation and Occupational Medicine of the Medical University of Vienna
- through scanning the QR codes that are printed on flyers which inform about the online video program or
- through opening the link printed under the QR code on the mentioned flyers.

## Results

The webcasts are structured as follows:

- In opening videos specialists in the field of hematology, pediatrics and physical medicine and rehabilitation speak about hemophilia and exercise from their perspective
- An introductory video informs about important preconditions such as sufficient factor protection and approval for the training and supervision through specialists (in the field of hematology, pediatrics, internal medicine, orthopedics or physical medicine and rehabilitation). Patients are also informed about contraindications for the training and receive instructions in the event of bleeding.
- A video regarding the composition of the exercises explains how to choose the appropriate exercises and how to increase the training with improving fitness. Here, general recommendations are given. These recommendations may be individually adapted according to the joint condition and the individual fitness and needs by specialists who treat the patient.
- The exercise videos contain exercises for warm up and mobilization, coordination, strengthening and flexibility. As each webcast can be accessed separately, each contains a short disclaimer (repeating the most important precautions and pointing to the introduction and exercise composition webcasts). The disclaimer is followed by instructions regarding the specific motor skill and by the exercise itself. In those webcasts that offer variations or advanced exercises, the respective videos are accessible one after the other as part of the same webcast by selecting the appropriate slide in the navigation bar. The exercises are demonstrated in the video, a physician comments on the exercises and points out what to pay attention to when performing the training.

Table 1 presents an overview of the exercise program. It contains exercises for warm up and mobilization, coordination, strengthening and flexibility.

**Table 1:** Overview of the exercise program

	Warm up and mobilization	Coordination exercises	Strengthening exercises for:	Flexibility exercises for:
Adults	Warm up and mobilization for ankles, knees, hips, spine, shoulders, elbows, wrists (sitting and standing position)	Standing with feet in narrow position; standing and walking in tandem position (increased difficulty: including the use of a ball); one leg stand (increased difficulty: including the use of a ball); one leg stand—performing movements like a “8” with the other leg	Back muscles, abdominals, abductors, adductors, quadriceps, tibialis anterior, calves, triceps/pectoralis/muscles of the shoulder	Iliopsoas, hamstrings, calves, quadriceps, pectoralis muscles/ventral muscles of the shoulder
Children, adolescents	Short program for the whole body while standing/walking on the same spot	One leg stand (increased difficulty: as described above); diagonal standing scale	Back muscles, abdominals, abductors, quadriceps, tibialis anterior, calves, triceps/pectoralis/muscles of the shoulder	Iliopsoas, hamstrings, calves, quadriceps, pectoralis muscles/ventral muscles of the shoulder

As the primary aim is to offer an exercise program to better stabilize the joints, training is focused on warm up and coordination, strength and flexibility exercises. Endurance training and the participation in sports are also recommended in PwH, but a detailed instruction on the choice of sports and the risk assessment of the different types of sport would have gone beyond the scope of the online training program. Regarding endurance training and sports, PwH are asked to follow the recommendations of their specialists in charge. Specific recommendations in this respect can be found in the pertinent literature [1, 47–57].

The training should to be increased over time according to the improving fitness, which is why the presented coordination and strengthening exercises can be adapted in difficulty, intensity and volume. Coordination exercises can thereby be increased as soon as an exercise can be safely conducted. Strengthening exercises should be increased in consultation with the specialist in charge and in the following order:

- Increase the number of repetitions: starting with only few repetitions (to learn the motion sequence appropriately), then increasing up to 20–25 repetitions
- Increase the load/weight (increase the load of the theraband)
- Increase the range of motion
- Increase the number of sets performed (up to three sets)
- Finally, an exercise of higher difficulty can be chosen. When doing so, it is recommended to start again with a low number of repetitions, in slow motion and a small range of motion and increase as given above.

Coordination and strengthening exercises should not be increased both at the same time but one after the other. In the case of swelling, pain or symptoms of a bleeding, the training should be stopped and a physician should be consulted. The training composition differs between the children and adult training program and is explained in the webcast on how to compose and increase the training. Adults are advised to focus their training on the chronically affected joint. In children who have not yet developed arthropathic joints, the training is recommended to strengthen the main deficits in children, which are coordination, flexibility of the back side of the legs and the strengthening of the hip abductors and abdominals [16, 32, 33, 48, 57, 58]. If children and adolescents already suffer from chronic joint affections, they are asked to consult their physician in order to focus the training on the affected joint similarly to the adult recommendations. In order to suggest a training program that is easily transferrable into everyday life, it is recommended to start with a training that involves an appropriate warm up and mobilization and 6 exercises consisting of coordination, strengthening and flexibility exercises to be performed 2–3 times a week on a regular base. The training can be extended after consultation with the physician in charge.

Both adults and children are advised to warm up and mobilize the joints and the spine before starting the training. It is especially important for adults to warm up appropriately with a focus on the chronically affected joints and the structures that are to be trained afterwards. Adults should take a minimum of 10 minutes for warm-up and mobilization before starting the actual training. Thereby, structures are prepared for the training, the risk of injury is reduced, and the joint range of motion is maintained. Also, PwH are advised to make use of the joint range of motion that is possible but to stay in the painless range.

Coordination exercises are important to improve balance and movement patterns and reduce the risk of injury. It is recommended to perform coordination exercises at the beginning of the training after warming up when the person is still in a resting state. In coordination as well as in strengthening exercises the main focus should rest on improving body awareness and increase the neural control of the movement according to the integrated model of joint function modified by Herbsleb et al. [59–63], whereby a slow, focused and concentrated training is beneficial.

Strengthening exercises aim to stabilize the joints and the spine. Sufficient warm up before the training is important. As already mentioned, a slow, focused and concentrated training, starting in a small range of motion, is recommended. To properly learn the movement, few repetitions are suggested to start until the exercise can be performed confidently. One repetition should last 5–7 seconds. PwH are advised to keep breathing steadily and not to hold the breath. If the exercise is already learned properly, adults can exhale during effort and inhale during relaxation.

Flexibility exercises help to maintain or improve the joint range of motion and reduce the risk of muscle shortening/contractures. Adults should stretch after performing strengthening exercises at the end of the training, children should ideally stretch before and – if there is enough time – also after performing strengthening exercises. Stretching should be done equally on both sides with 3 repetitions per exercise in a pain-free range, each stretch lasting approximately 20–30 seconds. In general, all exercises should only be practised when they can be performed without pain. General recommendations regarding the composition of the 6 exercises are given in Table 2.

**Table 2:** General recommendations regarding the exercise composition

Motor skill	Adults	Children, adolescents
Warm up, mobilization	≥10 min	Approx. 5 min
Coordination	1–2 coordination exercises	2 coordination exercises
Strengthening	2–3 strengthening exercises – chronic affection of knee: quadriceps (+ hip abductors, possibly with tibialis anterior) – chronic affection of ankle: tibialis anterior (+ quadriceps, possibly with calves) – chronic affection of elbow: triceps follow that scheme for 6–8 weeks, then keep focus on main muscle (quadriceps for knee, tibialis anterior for ankle, triceps for elbow) and vary by adding 1–2 other muscle groups for 6–8 weeks	2 strengthening exercises – 1 for abdominals – 1 for hip abductors – possibly: with back muscles follow that scheme for 6–8 weeks, then keep focus on abdominals and hip abductors and add exercises for the back muscles (quadriceps, tibialis anterior) for another 6–8 weeks
Flexibility	2 flexibility exercises – chronic knee affection: include stretching for hamstrings – chronic ankle affection: include stretching for calves	2 flexibility exercises – 1 for hamstrings – 1 for calves – if time: add 1 for iliopsoas

To start, the training composition is given in order to help PwH focus on the areas that may be the most effective in improving function and joint stability over the first 6–8 weeks; however, the recommendations can always be and should be individualized by the physician in charge. Over time, PwH are asked to vary the training by keeping the focus on one or two strengthening exercises as recommended to start with (adults: focussing on the chronically affected joint, children: primarily strengthening the hip abductors and abdominals) and in addition add one or two strengthening exercises for other muscle groups and keep that scheme for another 6–8 weeks. Coordination and strengthening exercises should be increased as given above according to the increasing fitness.

In order to take account of international recommendations to individualize the training for PwH according to the individual’s fitness, joint status and needs [1, 50], the Department of Physical Medicine, Rehabilitation and Occupational Medicine will offer physical medicine consultation hours for PwH. In this setting, PwH will receive individual recommendations regarding the composition and increase of the training, following a physical examination and additional radiological or functional diagnostical evaluation as needed. PwH can thereby also receive one-on-one physiotherapy, hemophilia-adapted group therapy, aquatherapy or bicycle ergometry as well as relaxation methods in a group setting or individually through biofeedback. Additionally, the physician can recommend certain modalities of physical therapy to be carried out at an institute close to where the patient lives or at the clinic (e.g. electrotherapy, ultrasound, laser, pulsed electromagnetic fields, massage) to reduce pain and improve function. The consultation hour will be offered once a month, a referral from a specialist is needed to schedule an appointment. For PwH living in and around Vienna, the appointment at the Department of Physical Medicine, Rehabilitation and Occupational Medicine will presumably go well together with the necessary follow-up appointments at the Department of Haematology or Pediatrics and thereby ideally complements the training offer for PwH.

## Discussion

In German-speaking countries attempts have already been made to educate PwH in multimodal disease-specific exercises and offer instructions to continue with a home-based training throughout the year. Sport camps take place once or twice a year and last for several days which are organized by the Austrian Haemophilia Society and the Interdisciplinary Center for Motion and Sports Medicine Wuppertal in cooperation with the University of Wuppertal in Germany and Switzerland [64, 65]. For children and adolescents in Germany, the Institute for Prevention and Aftercare (IPN) in Cologne offers so-called ‚Fit for Life‘ events. At these events, PwH receive an orthopedic joint examination and are tested regarding their physical fitness, motor skills and functional impairment. Depending on the findings, the boys then receive individual advice on home-based training and the choice of sports, an app helps children and their parents and teachers to choose appropriate school sports. Some of the recommended exercises at these events are also available at the given homepages [58, 65].

The online videos presented above aim to additionally support PwH with a regular home-based training. They also offer basic information on how to start training after being evaluated through specialists regarding sufficient factor therapy and contraindications for participating in the program. Data regarding the acceptance of the online program will be collected and presented at a later date.

Earlier studies concluded that exercise interventions for PwH are associated with specific challenges, such as the risk of injury, overload and potential bleeding. However, well-managed participation in exercise with adequate factor therapy and with respect to the individual’s joint status is known to improve a number of physical parameters [18, 36, 50, 66]. Regarding the safety of exercise interventions in PwH, a Cochrane review including eight randomized controlled trials assessed various training interventions in children and adults and found no training-induced adverse effects or bleedings [2]; however, regarding to the review’s authors, the safety of the applied interventions for severely affected patients remains unclear as some study groups used prophylactic factor prior to exercise and others included only subjects with moderate hemophilia [2]. It is especially important to be cautious when training is applied in patients with severe hemophilia, limited factor supply [2] or insufficient factor therapy, in patients with an inhibitor (an antibody directed against infused factor that inhibits the function of the factor) and with certain comorbidities. Adequate factor therapy can be a limiting factor for exercise in several countries [2].

Regarding strength training, static, dynamic and isokinetic exercises can be a part of a rehabilitation program for PwH as also recommended by Pietri et al. [31]. Exercising with relatively maximum resistance loads and roughly 6–10 repetitions to increase muscle mass and peak strength in healthy adults would cause a substantially higher risk of injury in PwH [38]. To strike a balance between strength improvement and a low risk of joint injury, several authors suggested reducing the risk by initially learning to use the accurate technique, training at submaximum loads, at a lower velocity, in limited joint ranges, or even isometrically at various joint angles [38, 67–72]. Therefore, the recommendations regarding strength training focus on slow repetitions and a concentrated performance. The authors advise starting with a low number of repetitions in a limited range of motion and increasing the training as ability improves. These recommendations are consistent with Negrier et al. [50].

The recommendations presented in the videos can always be adapted and increased individually by the physician in charge. According to the literature, strength training recommendations should be used as a guide only. The appropriate exercise should always be adapted to the individual’s needs and should be prescribed by healthcare professionals trained in hemophilia care who ensure the appropriateness, sufficient factor therapy and protective gear if necessary [1, 50]. Therefore, each webcast points to the necessity of a sufficient factor protection and an approval of the training through specialists prior to starting the training (hematologist, pediatrician, specialist for internal medicine, orthopedist or a specialist in physical medicine and rehabilitation) and highly recommends an ongoing supervision throughout the training. For PwH living in and around Vienna, the physical medicine consultation hour at the Department of Physical Medicine, Rehabilitation and Occupational Medicine at the Medical University of Vienna offers an individual adaption and supervision of the exercises.

The cited Cochrane review on the safety and effectiveness of exercise in PwH assessed many study interventions that trained several motor skills [2]. Regarding the effectiveness of the included training interventions, the authors found that in most exercise interventions one or more of the measured outcome parameters including pain, range of motion, strength and walking tolerance improved [2]. Functional exercises, such as treadmill walking and partial weight bearing exercises seemed to be more effective than static or short arc exercises regarding improvements in muscle strength [2]. The findings were consistent with several non-controlled intervention reports in the haemophilia literature [2].

According to the American College of Sports Medicine (ACSM) guidelines for resistance training for healthy adults, dynamic training with intensities of at least 40–50% of the one-repetition maximum (or the appropriate correlate in maximum isometric force) with ≤ 15 repetitions is required to improve muscle strength. The existing literature suggests that in children and adults with hemophilia not only training in intensities according to the ACSM resistance exercise criteria but also low resistance dynamic, isokinetic and partly also isometric combined with dynamic training of sufficient frequency and duration seem to be able to increase muscular strength [17, 18, 37, 73–79]. Regarding training frequency, ACSM guidelines for healthy adults recommend to train 2–3 times a week with a rest period to promote cellular adaptations [34]. In PwH, the training should be performed in accordance with the factor replacement regimen and the individual recovery time needed.

## Conclusion

Increased physical fitness is known to have multiple benefits in PwH. Sufficient strength, flexibility and coordination are important to improve joint stability and function, increase bone mass and reduce the risk of injury which might possibly reduce the risk of bleeding. Therefore, a regular multimodal training is important for PwH. As the distance to hemophilia treatment centers with professionally guided exercise training may be a challenge for PwH who live too far away to participate regularly, the online video program was implemented to close the gap and offer easily accessible and free of charge training recommendations that can be applied at home on a regular base. It is important that the patients participating in the training are under sufficient factor therapy according to their hematologist or pediatrician. To ensure safety, the training should be prescribed and supervised through musculoskeletal specialists with experience in the field of hemophilia and should be adapted individually according to the individual’s needs. One possibility to do so is the consultation hour at the Department of Physical Medicine, Rehabilitation and Occupational Medicine at the Medical University of Vienna. Further information and access to the online videos is available through the clinic’s homepage: http://www.meduniwien.ac.at/physmedrehab-videos*.*

### Implications for future research

Data regarding the acceptance and utilization of the online video program will be collected and presented at a later date.
